# Understanding cause of stillbirth: a prospective observational multi-country study from sub-Saharan Africa

**DOI:** 10.1186/s12884-019-2626-7

**Published:** 2019-12-04

**Authors:** Mamuda Aminu, Sarah Bar-Zeev, Sarah White, Matthews Mathai, Nynke van den Broek

**Affiliations:** Centre for Maternal and Newborn Health, Liverpool School of Tropical Medicine, Pembroke Place, Liverpool, L3 5QA UK

**Keywords:** Stillbirth, Cause of stillbirth, Asphyxia, Perinatal death audit, Quality of care, Sub-Saharan Africa, Low- and middle-income countries

## Abstract

**Background:**

Every year, an estimated 2.6 million stillbirths occur worldwide, with up to 98% occurring in low- and middle-income countries (LMIC). There is a paucity of primary data on cause of stillbirth from LMIC, and particularly from sub-Saharan Africa to inform effective interventions. This study aimed to identify the cause of stillbirths in low- and middle-income settings and compare methods of assessment.

**Methods:**

This was a prospective, observational study in 12 hospitals in Kenya, Malawi, Sierra Leone and Zimbabwe. Stillbirths (28 weeks or more) were reviewed to assign the cause of death by healthcare providers, an expert panel and by using computer-based algorithms. Agreement between the three methods was compared using Kappa (κ) analysis. Cause of stillbirth and level of agreement between the methods used to assign cause of death.

**Results:**

One thousand five hundred sixty-three stillbirths were studied. The stillbirth rate (per 1000 births) was 20.3 in Malawi, 34.7 in Zimbabwe, 38.8 in Kenya and 118.1 in Sierra Leone. Half (50.7%) of all stillbirths occurred during the intrapartum period.

Cause of death (range) overall varied by method of assessment and included: asphyxia (18.5–37.4%), placental disorders (8.4–15.1%), maternal hypertensive disorders (5.1–13.6%), infections (4.3–9.0%), cord problems (3.3–6.5%), and ruptured uterus due to obstructed labour (2.6–6.1%). Cause of stillbirth was unknown in 17.9–26.0% of cases.

Moderate agreement was observed for cause of stillbirth as assigned by the expert panel and by hospital-based healthcare providers who conducted perinatal death review (κ = 0.69; *p* < 0.0005). There was only minimal agreement between expert panel review or healthcare provider review and computer-based algorithms (κ = 0.34; 0.31 respectively *p* < 0.0005).

**Conclusions:**

For the majority of stillbirths, an underlying likely cause of death could be determined despite limited diagnostic capacity. In these settings, more diagnostic information is, however, needed to establish a more specific cause of death for the majority of stillbirths. Existing computer-based algorithms used to assign cause of death require revision.

## Background

Every year, an estimated 2.6 million stillbirths occur worldwide, with up to 98% occurring in low- and middle-income countries (LMIC) [1]. While the average stillbirth rate (SBR) in high-income countries is 3 per 1000 births (2–5 per 1000), the rates observed in many settings in sub-Saharan Africa and Southern Asia are up to 10-fold higher [1].

Most stillbirths in LMIC are considered to be preventable through provision of quality care for all mothers and babies [2–5]. The World Health Assembly (2014) endorsed a new global target: to reduce the stillbirth rate to 12 or fewer stillbirths per 1000 births in every country by 2030, providing a much-needed global target for reducing the burden of stillbirths [6].

To be effective, interventions to reduce stillbirths need up-to-date data about cause of stillbirth. However, there is a paucity of primary data on cause of stillbirth from LMIC, and particularly from sub-Saharan Africa. The most-relied upon sources of national data, such as the Demographic and Health Surveys, are limited in scope and do not enable examination of cause of stillbirth [7]. In an earlier systematic review of 142 papers on cause of, and factors associated with, stillbirth in LMIC [8], only about one-third of the included studies came from sub-Saharan Africa, even though the region has the highest stillbirth rates and the slowest rate of progress [1]. Most of the studies included in the review were single-hospital studies and many were narrowly focused on only a few specific probable causes of death. Thus, both researchers and implementers currently rely on limited and often outdated information to plan and execute programmes aimed at reducing preventable stillbirths.

Perinatal audit or review is an effective and evidence-based method which enables healthcare providers to collate information on cases of stillbirth and neonatal death [9, 10], review this information to understand the cause of, and factors contributing to, death and to formulate recommendations for change in practice. Actions taken to improve quality of care following perinatal death audit could potentially reduce perinatal mortality by as much as 30% [11]. Such reviews can be undertaken by trained assessors who make up an external expert panel or more commonly are conducted by health providers themselves with cases presented, for example, at monthly audit meetings.

Maternal death audit or review is already established and ongoing in many countries. However, perinatal death (especially stillbirth) reviews are less commonly conducted. The sheer number of stillbirths which occur can be overwhelming. Also, there are a fairly diverse range of classification systems in place which can make it difficult to assign cause of death when there is limited diagnostic capacity and/or healthcare provider knowledge and understanding of aetiology of disease [12]. Computer algorithms have the potential to reduce bias, and make the review process more transparent and consistent, faster and easier especially in settings with untrained staff and high stillbirth rates. Computer-generated hierarchical algorithms have been developed and used to assign cause of stillbirths in a community-based study [13]. However, they used verbal autopsy data, and it is unclear how the algorithms perform using hospital records or when compared with healthcare provider or expert panel review.

This study was conducted to investigate cause of stillbirth across four countries in sub-Saharan Africa. Three methods of assessment of cause of death were compared including i) review by healthcare providers, ii) review by an expert panel, and, iii) use of a new set of computer-based algorithms to determine cause of stillbirth. The performance of computer-based algorithms in assigning cause of death was assessed.

## Methods

### Study setting and design

Details have been published elsewhere [14]. Briefly, this was a prospective observational study in 12 hospitals, all of which were designated to provide comprehensive emergency obstetric care. The hospitals were located in Kenya (3), Malawi (4), Sierra Leone (2) and Zimbabwe (3). All were participating in a programme to support perinatal death review and were purposively selected because of the high number of births (at least 2000 births per year). In each healthcare facility, a team of four to eight healthcare providers (nurse-midwives and doctors) were trained to conduct perinatal death audit.

### Study population and sample size

The total number of births, live births and stillbirths were obtained monthly from existing healthcare facility registers (labour ward, discharge and theatre registers). A stillbirth was defined as a baby born without any sign of life at 28 weeks of gestation or more, or with a birthweight of 1000 g or more [15].

All stillbirths were identified sequentially until a predetermined sample size of 279 per country was reached. With this sample size if the proportion with a given cause was 24% the margin of error would be 5% using the 95% confidence level. In each country, the sample to be achieved was divided between the hospitals based on the number of births expected in each hospital. For the purpose of this study, data collection was discontinued when the predetermined sample size in each country was reached.

### Data collection

On a monthly basis (2014–2015), healthcare providers in each of the participating hospitals reviewed all stillbirths that had occurred in the preceding month. Information was extracted from case records and hospital registers using a pre-designed data collection form. Data collected included date of birth, maternal sociodemographic characteristics, pregnancy details, obstetric and medical history, baby’s characteristics (sex, weight, physical appearance), documented cause of death and factors that might have contributed to the death. Other variables required for use as denominators in calculating rates (total births and total live births) were obtained from labour ward and theatre registers. No specific diagnostic screening was possible or had been conducted in participating hospitals.

### Development of algorithms

In the first instance, a hierarchical list of the 37 most common causes of perinatal mortality was compiled from the literature [16, 17]. For each possible cause on the list, a rating was assigned (1–37) with asphyxia considered the most likely underlying cause of death (1) and unknown as the least likely (37) (Supplementary File 1). For each possible cause of death, a combination of clinical symptoms, signs and results of laboratory investigations to support the diagnosis for each of the possible most likely causes of death was created to form the initial algorithms. These algorithms were then reviewed by 155 experts in maternal and newborn health (obstetricians, nurse-midwives, paediatricians, public health researchers and general medical practitioners) participating in two international conferences. Based on their feedback, the algorithmic combinations of symptoms for individual diagnosis were improved and subjected to further review by experts in feto-maternal medicine (five obstetricians, two paediatricians and a midwife). These were subsequently imported into Excel Macro (Microsoft®, 2016).

### Assigning cause of death

Cause of death for each stillbirth was assessed separately using each of three different methods:

i. Healthcare providers: Each case was reviewed in detail by the team of healthcare providers working in each participating hospital and the most likely cause of death was agreed based on available information and by reaching consensus.
ii. Expert panel: The completed data extraction forms were separately reviewed by an expert panel consisting of eight experts in maternal and newborn health with experience in LMIC (midwives, doctors, obstetricians and a paediatrician). Each case was reviewed by at least one expert who independently assigned the most likely cause of death. One-quarter of the sample (*n* = 324) was randomly selected for review by a second expert reviewer. The proportion of cases for second review was calculated using Epi Info® (Version 7.2.0.1; CDC, 2016), by assuming 50% expected frequency of disagreement (to yield maximum sample) at 95% confidence level. This yielded 295, but an additional 29 cases were included in case of possible case exclusions.
iii. Computer algorithms: The data obtained from the field for each stillbirth was entered into an Excel spreadsheet and the algorithms (as developed above) were applied and used to assign cause of death.

### Data analysis

Antepartum stillbirth was defined as a macerated stillbirth whose mother arrived at the hospital without a fetal heart sound or a macerated stillbirth whose fetal heart sound was not documented on labour admission (Table 1). An intrapartum stillbirth was defined as a fresh stillbirth or a stillbirth whose fetal heart sound was detected and documented during labour irrespective of the physical appearance of the baby at birth. Stillbirths that could not be categorised as either antepartum or intrapartum stillbirths were designated as unspecified.

**Table 1:** Criteria used to determine time of death based on physical appearance of the baby at birth and presence or absence of fetal heart sound at time of admission

Fetal Heart Sound on Labour Admission	Appearance		
	Fresh Stillbirth	Macerated Stillbirth	Unspecified
Present	Intrapartum Stillbirth	Intrapartum Stillbirth	Intrapartum Stillbirth
Absent	Intrapartum Stillbirth	Antepartum Stillbirth	Unknown Time of Death
Unknown	Intrapartum Stillbirth	Antepartum Stillbirth	Unknown Time of Death

Cause of stillbirth obtained by each of the three methods was compared. Descriptive analyses were conducted using SPSS® (IBM, NY, version 22), with 95% confidence intervals (CI) where appropriate. Kappa (κ) analysis was used to compare the cause of death assigned using each of the three methods (i to iii above). To enable this, causes of stillbirth assigned were grouped using the Classification of Stillbirth by Relevant Condition at Death (ReCoDe), which was selected for its simple structure and manageable number of categories [16]. Kappa scores were interpreted using a modified Cohen’s convention: no agreement (0 to 0.2), minimal (0.21 to 0.39), weak (0.40 to 0.59), moderate (0.60 to 0.79), strong (0.80 to 0.90) and almost perfect agreement (above 0.90) [18].

## Results

There were 1563 stillbirths recorded among 43,979 births in the 12 selected hospitals. Data collection was stopped when the sample size of 1329 cases was reached. On review, 1267 (95.3%) met the definition for stillbirth stipulated for this study and were included in the analysis; 321 in Kenya, 299 in Malawi, 340 in Sierra Leone and 307 in Zimbabwe (Table 2).

**Table 2:** Demographic and clinical characteristics of study population (*n* = 1267)

Characteristics	Kenya *n* = 321% (n)	Malawi *n* = 299% (n)	Sierra Leone *n* = 340% (n)	Zimbabwe *n* = 307% (n)	Total *n* = 1267% (n)
Maternal age (years)					
< 15	0.0 (0)	1.0 (3)	0.3 (1)	0.0 (0)	0.3 (4)
15–19	9.1 (29)	16.0 (47)	24.2 (81)	18.8 (33)	15.2 (190)
20–24	28.6 (91)	31.3 (92)	22.4 (75)	24.6 (75)	26.6 (333)
25–29	30.8 (98)	21.1 (62)	23.6 (79)	25.9 (79)	25.4 (318)
30–34	16.0 (51)	15.3 (45)	14.3 (48)	20.3 (62)	16.4 (206)
35–39	10.7 (34)	9.2 (27)	9.9 (33)	11.5 (35)	10.3 (129)
> = 40	2.5 (8)	3.4 (10)	2.1 (7)	3.6 (11)	2.9 (36)
No information	2.2 (7)	2.7 (8)	3.3 (11)	3.3 (10)	2.9 (36)
Mean (SD)	26.6 (5.8)	25.7 (6.6)	25.2 (6.4)	27.2 (6.5)	26.2 (6.4)
Parity					
Para 1	31.8 (101)	33.3 (98)	29.9 (100)	34.1 (104)	32.2 (403)
Para 2–4	61.0 (194)	54.8 (161)	50.7 (170)	63.0 (192)	57.2 (717)
Para 5 or more	5.0 (16)	10.2 (30)	17.3 (58)	2.6 (8)	8.9 (112)
Mothers’ place of residence					
Urban	19.2 (61)	42.9 (126)	46.6 (156)	84.6 (258)	48.0 (601)
Semi-urban	24.5 (78)	8.5 (25)	1.8 (6)	3.0 (9)	9.4 (118)
Rural	53.8 (171)	39.1 (115)	49.9 (167)	11.5 (35)	38.9 (488)
No information	2.5 (8)	9.5 (28)	1.8 (6)	1.0 (3)	3.6 (45)
Antenatal care					
At least 1 visit	85.5 (272)	68.7 (202)	43.3 (145)	72.8 (222)	67.1 (841)
4 or more visits	34.9 (111)	9.9 (29)	1.2 (4)	24.6 (75)	17.5 (219)
Referral status					
Referral from other facility	37.1 (118)	33.0 (97)	40.6 (136)	69.5 (212)	44.9 (563)
Came from home	61.3 (195)	63.9 (188)	58.2 (195)	28.5 (87)	53.1 (665)
No information	1.6 (5)	3.1 (9)	1.2 (4)	2.0 (6)	1.9 (24)
Type of pregnancy					
Singleton	91.6 (294)	90.0 (269)	88.8 (302)	92.5 (284)	90.7 (1149)
Multiple	6.2 (20)	9.7 (29)	7.4 (25)	4.9 (15)	7.0 (89)
Gestational age at delivery					
28 to 31 completed weeks	21.8 (70)	8.0 (24)	8.5 (29)	20.5 (63)	14.7 (186)
32 to 36 completed weeks	26.5 (85)	23.4 (70)	27.6 (94)	33.2 (102)	27.7 (351)
37 completed weeks or more	46.1 (148)	59.9 (179)	61.5 (209)	40.7 (125)	52.2 (661)
No information	5.6 (18)	8.7 (26)	2.4 (8)	5.5 (17)	5.5 (69)
Mode of delivery					
Spontaneous vaginal birth	71.7 (230)	64.6 (192)	66.8 (227)	71.0 (218)	68.4 (867)
CS (includes laparotomy for ruptured uterus)	26.2 (84)	30.8 (92)	29.1 (99)	28.7 (88)	28.7 (363)
Instrumental delivery	0.3 (1)	3.0 (9)	2.4 (8)	0.0 (0)	1.4 (18)
Destructive operation	0.0 (0)	1.3 (4)	0.0 (0)	0.0 (0)	0.3 (4)
No information	1.9 (6)	0.7 (2)	1.8 (6)	0.3 (1)	1.2 (15)

### Characteristics of the study population

The mean age of 1253 included mothers was 26.2 years (standard deviation (SD) 6.4), with only a slight variation between countries (Table 2). The mean gestation at birth (estimated mostly from the last menstrual period) was 35.8 weeks (SD 3.5). Only 54 cases (4.3%) had an ultrasound scan in early pregnancy for confirmation of gestational age. The majority were singletons (90.7%), while 7% were from multiple gestations. Spontaneous vaginal delivery accounted for 68.2% of stillbirths, while 303 (23.9%) were born by caesarean section. Sixty (4.7%) mothers underwent laparotomy for ruptured uterus. Four babies were born vaginally following a destructive procedure.

### Stillbirth rate and time of death

**Fig. 1:**
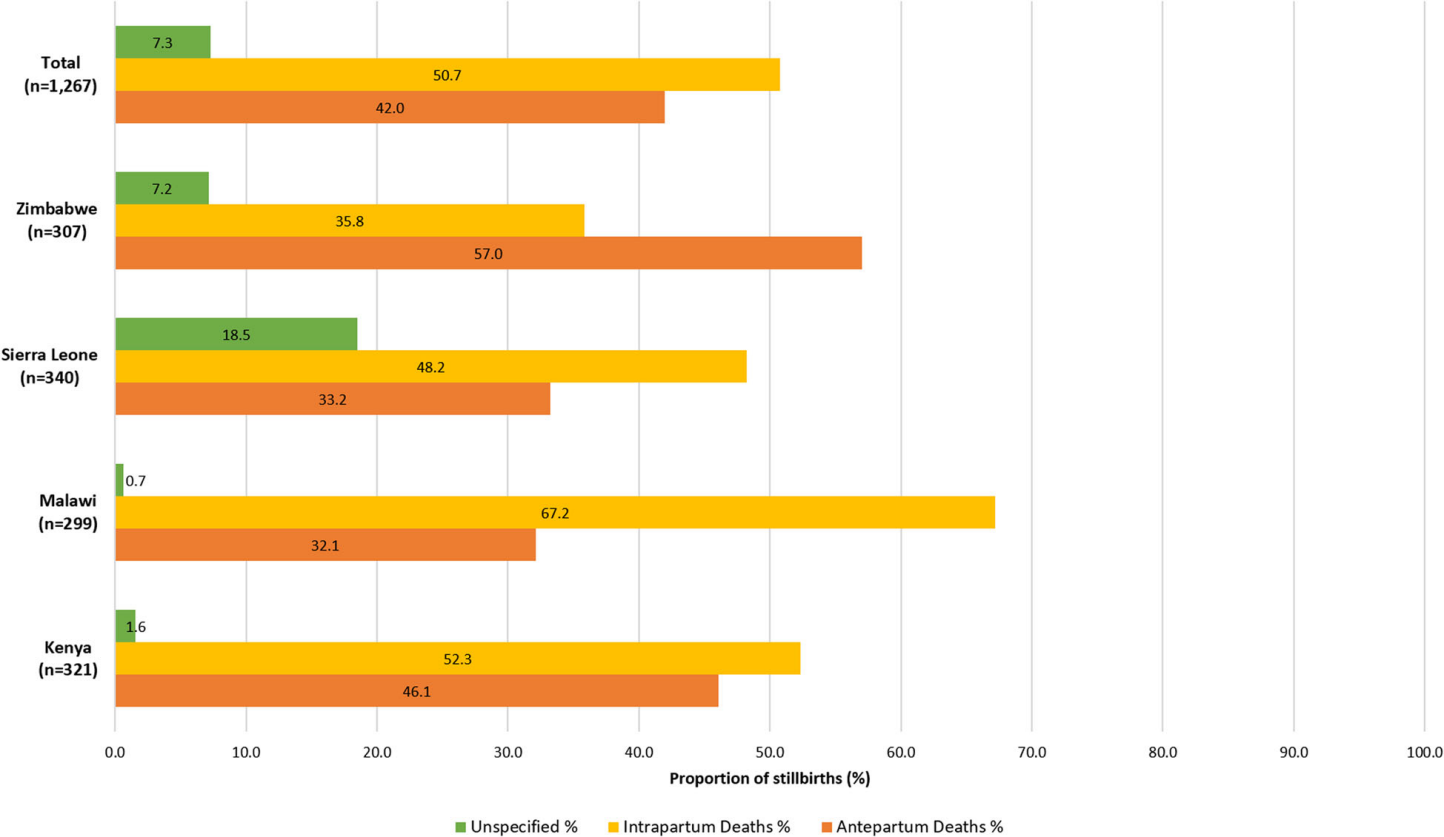
Proportion of antepartum and intrapartum stillbirths by country and for all stillbirths combined

The stillbirth rate varied among countries and was lowest in Malawi (20.3 per 1000 births; 95% CI: 15.0–42.8), followed by Zimbabwe (34.7 per 1000 births; 95% CI: 31.8–39.2), Kenya (38.8 per 1000 births; 95% CI: 33.9–43.3) and Sierra Leone (118.1 per 1000 births; 95% CI: 115.0–121.2). Of the 1267 cases, 35.9% were documented as fresh stillbirths. However, up to half (50.7%) of all cases met the study’s criteria for intrapartum deaths (Fig. 1). The highest proportion of intrapartum deaths was observed in Malawi (67.2%), and the lowest in Zimbabwe (35.8%).

### Cause of stillbirth

The proportion of all cases of stillbirth for which no cause could be established was highest when reviewed by the expert panel review (26.4%) and lowest when computer algorithms were applied (17.9%). In general, cause of death was more difficult to establish for antepartum deaths (with 29.5 to 36.8% recorded as unknown) than for intrapartum deaths (6.8 to 16.5% recorded as cause unknown).

The leading cause of stillbirth was reported to be birth asphyxia. The highest proportion of stillbirths due to asphyxia was assigned when using computer-based algorithms (37.4%), with much lower proportions obtained after healthcare providers’ (HCPs) review (18.5%) and expert panel review (20.4%) (Table 3; Additional file 1: Table S1). For stillbirths identified to have occurred intrapartum, the proportion considered to be due to asphyxia ranged from 26.6 to 69.4%, with the computer algorithms assigning the highest proportion to asphyxia.

**Table 3:** Cause of stillbirth by method of assessment for antepartum (ASB) and intrapartum stillbirth (ISB) and for all stillbirths combined

Method of Assessment	Healthcare providers’ review			Computer-based algorithms			Expert panel review		
Cause of Death	ASB^a^ *n* = 532% (n)	ISB^a^ n = 643% (n)	Total^b^ n = 1267% (n)	ASB^a^ *n* = 526% (n)	ISB^a^ *n* = 599% (n)	Total^b^ *n* = 1215% (n)	ASB ^a^ *n* = 532% (n)	ISB^a^ *n* = 643% (n)	Total^b^ *n* = 1267% (n)
Asphyxia	**9.6 (51)**	**26.6 (171)**	**18.5 (234)**	**6.3 (33)**	**69.4 (416)**	**37.4 (455)**	**10.3 (55)**	**29.7 (191)**	**20.4 (258)**
Placental disorders	**13.0 (69)**	**15.1 (97)**	**15.1 (191)**	10.6 (56)	5.2 (31)	8.4 (102)	**10.7 (57)**	**13.4 (86)**	**13.1 (166)**
Hypertensive disorders	**14.5 (77)**	**13.2 (85)**	**13.6 (172)**	8.7 (46)	1.5 (9)	5.1 (62)	**15.0 (80)**	**12.3 (79)**	**13.3 (169)**
Cord problems	5.3 (28)	7.9 (51)	6.5 (82)	2.9 (15)	3.3 (20)	3.3 (40)	4.5 (24)	7.8 (50)	6.2 (78)
Infectious conditions	10.0 (53)	4.4 (28)	6.6 (84)	**14.4 (76)**	**3.8 (23)**	**9.0 (109)**	7.7 (41)	2.8 (18)	4.7 (60)
Amniotic problems	3.9 (21)	3.9 (25)	3.7 (47)	–	–	–	3.4 (18)	3.7 (24)	3.4 (43)
Congenital anomalies	1.7 (9)	3.1 (20)	2.4 (31)	3.0 (16)	0.7 (4)	1.8 (22)	1.9 (10)	3.1 (20)	2.5 (32)
Ruptured uterus	2.6 (14)	7.0 (45)	5.2 (66)	2.9 (15)	1.7 (10)	2.6 (32)	3.0 (16)	7.8 (50)	5.7 (72)
Diabetes	0.9 (5)	0.3 (2)	0.6 (7)	0.2 (1)	0.2 (1)	0.2 (2)	0.6 (3)	0.3 (2)	0.4 (5)
Fetal growth restriction	–	–	–	**19.4 (102)**	**5.0 (30)**	**12.9 (157)**	0.4 (2)	0.6 (4)	0.5 (6)
Deep vein thrombosis	0.2 (1)	–	0.1 (1)	–	–	–	–	–	–
Iatrogenic	–	0.2 (1)	0.1 (1)	0.2 (1)	–	0.1 (1)	–	–	–
Post-maturity	–	0.2 (1)	0.1 (1)	–	–	–	–	–	–
Prematurity	5.8 (31)	1.4 (9)	3.3 (42)	–	–	–	3.4 (18)	0.5 (3)	1.7 (21)
Rhesus isoimmunisation	0.2 (1)	0.3 (2)	0.2 (3)	0.2 (1)	–	0.1 (1)	–	–	–
Sickle cell disease	0.2 (1)	0.2 (1)	0.2 (2)	–	–	–	–	–	–
Anaemia in pregnancy	1.7 (9)	1.4 (9)	1.4 (18)	–	–	–	0.6 (3)	0.5 (3)	0.5 (6)
Traditional abortive herbs	0.2 (1)	–	0.1 (1)	–	–	–	0.2 (1)	–	0.1 (1)
Trauma (external)	0.8 (4)	0.5 (3)	0.6 (7)	0.2 (1)	0.2 (1)	0.2 (2)	0.8 (4)	0.3 (2)	0.5 (6)
Birth trauma	–	–	–	–	–	–	0.8 (4)	0.8 (5)	0.7 (9)
Twin-twin transfusion	–	–	–	0.6 (3)	0.8 (5)	0.1 (12)	–	–	–
Unknown % (n)	29.5 (157)	14.5 (93)	21.9 (277)	31.6 (166)	6.8 (41)	17.9 (218)	36.8 (196)	16.5 (106)	26.4 (335)

Top 3 causes for each method of assessment highlighted in bold

^a^ *ASB* Antepartum stillbirth, *ISB* Intrapartum stillbirth, Total^b^ Includes cases with unknown time of death

The proportion of cases due to placental disorders (mainly placenta abruptio and praevia) also varied – this was most often identified as a cause of death by healthcare providers (15.1%). Similarly, the proportion of stillbirth considered to be related to hypertensive disorders (hypertension in pregnancy, pre-eclampsia and eclampsia) varied by the method of assessment, accounting for 5.1% using algorithms and up to 13.6% following healthcare provider review.

No amniotic conditions associated with stillbirth (such as oligo- and polyhydramnios) were identified using algorithms. On the other hand, more cases of stillbirth were recorded to be the result of fetal growth restriction and twin-twin transfusion using the application of algorithms, compared to healthcare provider or expert panel review. Prematurity and anaemia in pregnancy were not identified as direct causes of stillbirth using algorithms.

For antepartum deaths, hypertensive disorders and infections were among the leading causes identified, with minimal variations between healthcare providers and the expert panel.

A particularly high proportion of death due to fetal growth restriction was diagnosed when computer algorithms were applied.

**Fig. 2:**
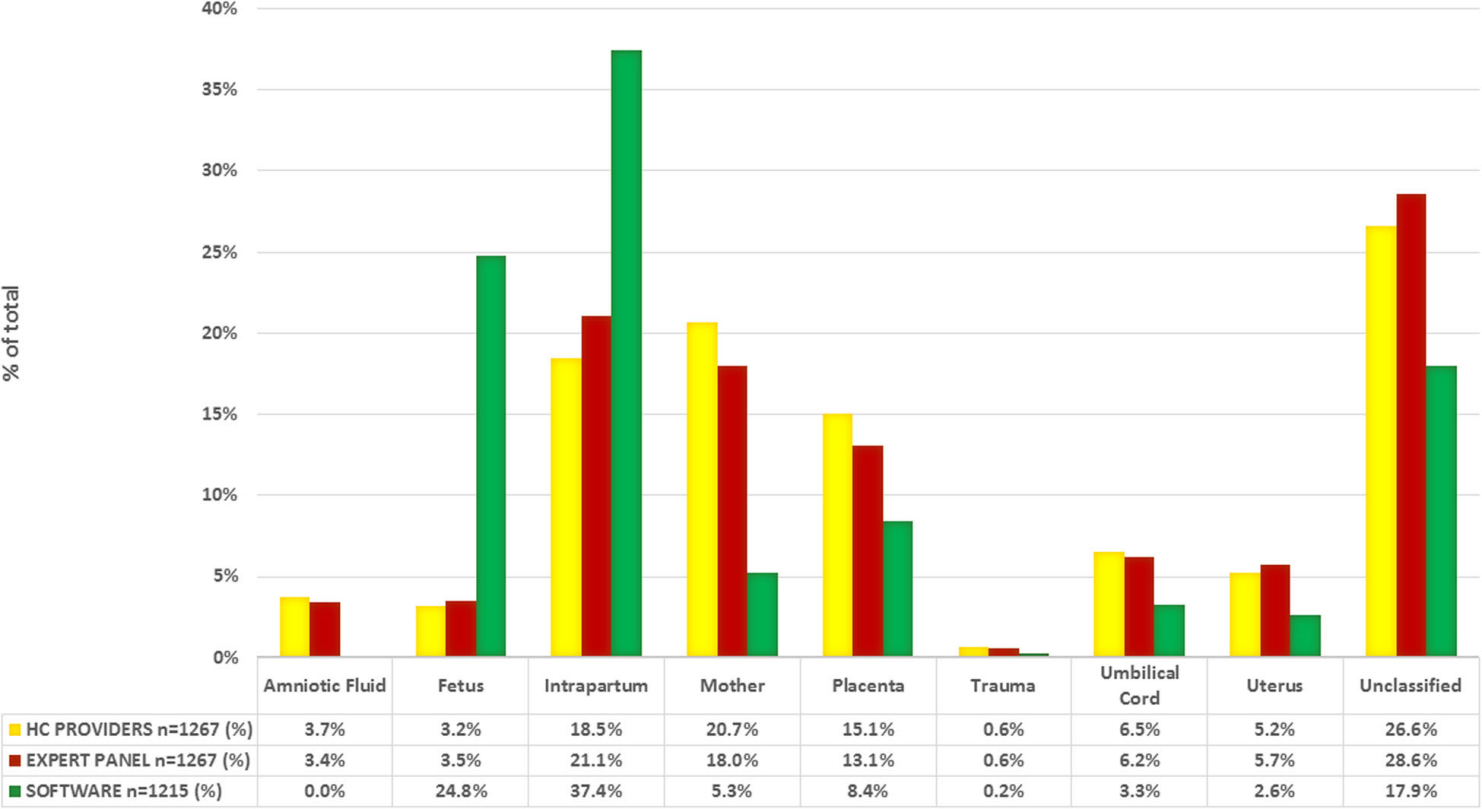
ReCoDe classification of cause of death by method of assessment

When the ReCoDe classification was applied to cause of death, categories for cause of death varied by methods used to assign cause of death (Fig. 2). For example, in the fetal category, the computer algorithms reported 24.8%, approximately seven-fold more than the result by expert panel (3.5%).

### Methods for assigning cause of stillbirth

Algorithms could not be used to assign cause of death for 52 cases due to missing data, and these were excluded. Kappa analysis of the ReCoDe data to explore the level of agreement between the three methods used to assign cause of death showed a moderate agreement between cause of death assigned by HCPs and that assigned by expert panel (κ = 0.69; *p* < 0.0005). The analysis between cause of death assigned by expert panel and cause assigned using computer-based algorithms showed a minimal agreement (κ = 0.34; *p* < 0.0005). Similarly, the results obtained after HCP’s review compared to the use of algorithms showed a minimal agreement (κ = 0.31; *p* < 0.0005).

Among the 324 cases that were randomly selected for a second expert review, inter-observer variations were observed in 91 cases (28.1%). The agreement rate between experts was only moderate (κ = 0.61; *p* < 0.0005), and lower than the level of agreement between the expert panel and the HCPs (κ = 0.69; *p* < 0.0005). Disagreement was highest for “unclassified” category (47% of the 91 cases), and lowest for fetal and amniotic causes (1% each) (Additional file 2: Dataset S1).

## Discussion

### Main findings

Stillbirth rates in participating healthcare facilities were high, ranging between 20.3 and 118.1 per 1000 births. Half (50.7%) of the 1267 stillbirths included in the analysis could be classified as intrapartum stillbirths.

Asphyxia was the most common cause of stillbirth reported overall irrespective of method of assessment of cause of death (range from 18.5–37.4%). For the group of intrapartum stillbirths only, this ranged between 26.6% (by healthcare providers) to 69.4% (by computer algorithms). Other causes of death identified were: placental disorders (ranged from 8.4–15.1%), hypertensive disorders (5.1–13.6%), infections (4.7–9.0%), cord problems (3.3–6.5%), ruptured uterus (2.6–5.7%). The proportion of cases where a cause could not be established also varied (17.9–26.4%). For antepartum deaths, hypertensive disorders and infections are the leading cause of stillbirth but for a significant population (up to 36.8%) of antepartum stillbirths, cause of death could not be assigned.

Healthcare providers working at hospital level in each country were able to assign a cause of death in most cases and the agreement rate with an international specialist expert panel was moderately good (κ = 0.69; *p* < 0.0005). Computer-based algorithms were easy to apply, but there was very poor agreement with either the expert panel (κ = 0.34; *p* < 0.0005), or healthcare providers’ review (κ = 0.31; *p* < 0.0005).

### Strengths and limitations

There is still a marked lack of primary data on cause of stillbirths from low- and middle-income settings. There are several methods that are being used to assign the cause of death. To the best of our knowledge, this is the first study that compares the three most commonly used methods globally.

However, with only half of all stillbirths occurring in health facilities in sub-Saharan Africa [1], hospital-based stillbirth studies only tell part of the story. Furthermore, the ability to identify a cause of death depended on information obtained from case notes and registers which were often incomplete, inaccurate or both. Currently, in most low- and middle-income settings, there are no specific diagnostic tests available or used to help establish cause of death. In most cases autopsy is not possible. Even in high-income countries, the acceptance rate for stillbirth autopsy remains low [19]. This makes it difficult to achieve consistency in diagnosis across multiple settings. While there was agreement between healthcare providers and the expert panel, the use of computer-based algorithms remains problematic. This is at least in part due to the lack of specific data required to inform each component of an algorithm and to reach certain diagnosis contributes but algorithms will need to be amended to improve the likelihood of correctly assigning a cause of death when applied.

Kappa analysis could not be run without grouping the causes of death to reduce the number of empty cells in cross-tabulation. Hence, the use of the ReCoDe classification system [16]. This might have exaggerated or diminished the agreement rates in some categories.

### Stillbirth rates

Hospital-based stillbirth rates across many low-resource settings continue to be high and vary, ranging from 6.1 in Peru [20] to 170 per 1000 births in a Nigerian hospital [3]. However, there is a paucity of contemporaneous primary data from sub-Saharan Africa to enable better comparisons.

For Sierra Leone, there were no hospital-based studies for comparison. While the national stillbirth rate of 8.1 per 1000 births is understandably lower in community surveys [21], the high hospital stillbirth rate reported in our study (118 per 1000 births; 95% CI: 115.0–121.2) could be partly explained by the period of data collection, which coincided with the Ebola virus outbreak in West Africa. Although hospitals included in this study did not treat confirmed cases of Ebola, at the time of the epidemic there was a remarkable reduction in the availability and access to maternal and newborn health services as staff and researchers were mobilised to deal with the epidemic. A 34% increase in facility maternal mortality ratio and 24% increase in stillbirth rates was observed across the facilities surveyed [22]. In one of the hospitals in this study, maternity services were only provided between morning and evening during the epidemic. Mothers in labour were discharged every evening and asked to come back the following morning if they had not given birth.

### Cause of stillbirth

The single most frequent cause of stillbirth was asphyxia, accounting for 18.5 to 37.4% of all stillbirths. However, in principle it can be argued that asphyxia is not a cause of death per se but the mode of the pathophysiological pathway leading to death. A study from six LMIC similarly reported that asphyxia was the leading cause of stillbirth accounting for 46.6% of 2847 stillbirths [13]. The variation may be attributed to the fact that McClure et al. used data from a population-based registry [13]; they also used a broader definition of stillbirth to include stillbirths which occurred from 20 weeks of gestation. To reduce stillbirths due to asphyxia, it is important to ensure care during childbirth is provided by skilled birth attendants including monitoring of fetal rate during labour and birth as recommended by the World Health Organization (WHO) [23]. This, could help in identifying where intervention is needed with an early response and action taken for conditions that can result in asphyxia.

Early detection of fetal growth restriction as a potential cause of stillbirth could reduce the proportion of stillbirths with unknown cause of death from 40 to 50% to less than 20% [16, 24]. A hospital-based study from Pakistan reported that fetal growth restriction accounted for 18% of all stillbirths [25], which is higher than the 12.9% found in this study. Interestingly, intrauterine growth restriction (IUGR) was not considered an underlying cause of stillbirth by either healthcare providers or the expert panel. Correct diagnosis of IUGR requires accurate information on gestational age and birthweight with standards adjusted for sex, birth order and ethnicity. Although the modelling used by computer algorithms corrects birthweight for gestational age, it should be noted that gestational age estimates are often unreliable or not available in resource limited settings [26]. In this study, assessment and documentation of gestational age was mostly based on reported last menstrual period and/or fundal height measurement during pregnancy.

Prematurity and anaemia in pregnancy were assigned as cause of stillbirth in some cases reviewed by HCPs and by the expert panel. However, these are not recognised as underlying cause of stillbirth per se, but rather understood to be factors associated with stillbirth. There is a variety of classification systems for stillbirth, with The WHO Application of ICD-10 to Deaths During the Perinatal Period (ICD-PM) [27] being a recent addition. Training is needed to help healthcare providers understand the aetiology of stillbirth and become conversant with the classification to be able to correctly and more frequently assign a likely cause of stillbirth.

### Methods of assigning cause of stillbirth

Differences in the assigned cause of death observed using each of the three methods emanated partly from prioritisation of certain diagnoses in the hierarchical model of the computer-based algorithms. In addition, specific information required when using the algorithms was often not available. For example, ultrasound evidence is required to make a diagnosis of oligo- or polyhydramnios which is part of the information required to be able to apply the algorithms. This was, however, rarely available. Variations in the proportion of stillbirths with an unknown cause are likely to reflect variations in capacity to make a diagnosis, lack of information and variation in contextual knowledge. Improving the quality and amount of clinical documentation would most likely reduce the proportion of stillbirths with unknown cause.

Perinatal death reviews conducted by HCPs allows for discussion and identification of common problems and identify where care needs to be improved and develop and implement with practical solutions to improve quality of care. Reviews by HCPs also provided opportunities for issues related to quality of care to be discussed in detail, for recommendations to be formulated, and to generate ideas for context-specific action plans. This is one of the main purposes of perinatal death review.

## Conclusion

Stillbirth rates are unacceptably high in LMIC. Asphyxia is the leading cause of stillbirth overall. Even with minimal information obtained from registers and case notes, healthcare providers in LMIC settings could identify a cause of stillbirth in most cases. Identifying cause of death can be difficult in low resource settings but this does form the basis of recommendation for changes in practice required to reduce preventable stillbirths. Improving the diagnostic work-up for stillborn babies could further reduce the proportion of stillbirths for which cause of death remains “unknown”. Computer-based algorithms could potentially be useful when large numbers of stillbirths need to be reviewed but will need the modifications to improve performance.

## Supplementary information

**Additional file 1: Table S1.** Possible cause of stillbirth ranked according to likelihood of occurrence as determined from the literature, and as used in the computer algorithms.

**Additional file 2: Dataset S1.** Cause of Death Agreement Rates

## Data Availability

The datasets used and/or analysed during the current study are available from the corresponding author on reasonable request.

## Abbreviations

CDC: Centre for Disease Control
CI: Confidence interval
HCPs: Healthcare providers
ICD-PM: The WHO application of ICD-10 to deaths during the perinatal period
IUGR: Intrauterine growth restriction
LMIC: Low- and middle-income countries (LMIC)
ReCoDe: Classification of Stillbirth by Relevant Condition at Death
SBR: Stillbirth rate
SD: Standard deviation
WHO: World Health Organization
κ: Kappa
