# Supplementary material for: Understanding cause of stillbirth: a prospective observational multi-country study from sub-Saharan Africa
Source: BMC Pregnancy Childbirth. 2019 Dec 4;19:470. doi: 10.1186/s12884-019-2626-7 (PMC6894270; doi:10.1186/s12884-019-2626-7)
Supplement: Supplementary file 1 — Additional file 1: Table S1. Possible cause of stillbirth ranked according to likelihood of occurrence as determined from the literature, and as used in the computer algorithms. [file 12884_2019_2626_MOESM1_ESM.docx]

**S1 Table 1: Possible cause of stillbirth ranked according to likelihood of occurrence as determined from the literature, and as used in the computer algorithms.**

| **CAUSE OF STILLBIRTH** | **RANK** |
| --- | --- |
| Asphyxia | 1 |
| Lethal congenital anomaly | 2 |
| Abruptio placentae | 3 |
| Ruptured uterus | 4 |
| Eclampsia | 5 |
| Pre-eclampsia | 6 |
| Cord prolapse | 7 |
| Gestational Hypertension | 8 |
| Chronic Hypertension | 9 |
| Syphilis | 10 |
| Placenta praevia | 11 |
| Diabetes | 12 |
| Chorioamnionitis | 13 |
| Oligohydramnios | 14 |
| Polyhydramnios | 15 |
| Fetal growth restriction | 16 |
| Twin-twin transfusion | 17 |
| Feto-maternal haemorrhage | 18 |
| Birth Trauma | 19 |
| Acute Infection | 20 |
| Malaria | 21 |
| HIV-Related complications | 22 |
| External trauma | 23 |
| Iso-immunisation | 24 |
| Placental insufficiency /infarction | 25 |
| Constricting loop or knot | 26 |
| Iatrogenic | 27 |
| Non-immune hydrops | 28 |
| Chronic Infection – e.g. TORCH | 29 |
| Vasa Praevia | 30 |
| Velamentous insertion | 31 |
| Thyroid diseases | 32 |
| Lupus/Antiphospholipid Syndrome | 33 |
| Cholestasis | 34 |
| Drug abuse | 35 |
| Unknown - Inadequate information available | 36 |
| Unknown - No relevant condition identified | 37 |
